# Chronic Pain in Adolescents: The Predictive Role of Emotional Intelligence, Self-Esteem and Parenting Style

**DOI:** 10.30476/ijcbnm.2020.83153.1129

**Published:** 2020-07

**Authors:** Maryam Shaygan, Zainab Karami

**Affiliations:** 1 Community Based Psychiatric Care Research Center, Department of Nursing, School of Nursing and Midwifery, Shiraz University of Medical Sciences, Shiraz, Iran; 2 Student Research Committee, Shiraz University of Medical Sciences, Shiraz, Iran

**Keywords:** Adolescents, Chronic pain, Emotional intelligence, Parenting style, Self-esteem

## Abstract

**Background:**

Pediatric chronic pain is prevalent and disabling. The present study aimed to assess the prevalence of chronic pain among adolescents in Shiraz, Iran. We also compared emotional intelligence, self-esteem and parenting style between adolescents with chronic pain and healthy adolescents. Finally, we examined the predicting role of these variables regarding chronic pain in adolescents.

**Methods:**

This cross-sectional study, from January to June 2018, was conducted on 734 adolescents in Shiraz. A clustering sampling method was used. Adolescents with chronic pain were identified by affirmative answers to screening questions based on the International Classification of Diseases 11th Revision (ICD-11) criteria. Participants completed three validated self-report questionnaires: Trait Emotional Intelligence Questionnaire, Rosenberg self-esteem scale and Baumrind parenting style questionnaire. The data were analyzed through SPSS v.22 software using Mann-Whitney and binary logistic regression tests. P<0.05 was considered significant.

**Results:**

There were 221(30.1%) adolescents who met the ICD-11 criteria of chronic pain. Mann-Whitney tests showed that emotional intelligence (P<0.001), self-esteem (P<0.001), authoritative parenting style (P=0.004), and authoritarian parenting style (P=0.006) were significantly different in adolescents with chronic pain compared to healthy adolescents. Binary logistic regression revealed that emotional intelligence (P<0.001), self-esteem (P<0.001), authoritarian parenting style (P=0.04) and authoritative parenting style (P=0.01) were significantly correlated with chronic pain after controlling for demographic variables.

**Conclusion:**

Our findings indicate that emotional intelligence, self-esteem and parenting styles could be important factors in development or maintenance of chronic pain in adolescents. The results have potential to be extended to future interventions for adolescents with chronic pain.

## INTRODUCTION

Chronic pain is one of the most important medical problems worldwide. It has numerous negative impacts on the individuals’ daily lives. ^1^ Chronic pain persists or recurs for longer than 3 months and is associated with significant emotional distress and functional disability. ^2^

One of the most important groups which may experience chronic pain is adolescents. Pediatric chronic pain is prevalent, disabling and costly. ^3^ Continuous experience of pain in adolescents leads to absence in school and avoidance of social activities. Adolescents with chronic pain cannot properly exercise or get involved in activities, and they are often depressed. ^1^ Moreover, pain during childhood and adolescence increases the risk of chronic pain during adulthood. ^4^ According to the biopsychosocial model of pain, a lot of psychological and social factors may influence the development of painful conditions. ^5^ Since chronic pain is common in adolescents and due to the widespread impacts of chronic pain on daily life as well as high costs of chronic pain, ^1 , 3 , 4^ it is necessary to identify psycho-social factors related with pain in adolescents.

One of the psychological factors studied in recent years is emotional intelligence. Emotional intelligence refers to the ability to identify and manage one’s own emotions, as well as those of others in order to be able to communicate with others effectively. ^6^ Having more emotional intelligence tends to be associated with better communication skills and less stress in adolescents. ^7 , 8^ Stress is well recognized as a risk factor of chronic pain in adolescents. ^9^ Therefore, it is hypothesized that adolescents with more emotional intelligence are protected from chronic pain due to less experience of stress. However, any definite comment about this matter needs more studies and investigations. 

Another factor which is related to adolescents’ physical and mental health is self-esteem. One recent study has shown that self-esteem is the most significant predictor for depressive and anxiety symptoms during early adolescence. ^10^ Since depression and anxiety are considered as the important predictive factors of pain, ^11^ it is possible that there is a negative association between self-esteem and chronic pain in adolescents. However, it is necessary to conduct more research about it.

Parenting style is another effective factor on adolescents’ mental health. ^12^ Baumrind identified three different patterns of parental authority, namely authoritarian, authoritative and permissive. ^13^ Authoritarian style is characterized by more demands and less responsiveness of parents. ^14 , 15^ Authoritative style presents appropriate levels of independence and mutual relationships between the child and parents by a combination of skills and high emotional support. ^14^ In permissive style which is characterized by less demand of parents and their more responsiveness, parents have less expectation of their children because of paying more attention to them. Previous studies have shown the beneficial impact of authoritative parenting and the negative impact of authoritarian parenting on children’s mental health. ^16^

Although previous research has focused on the relationship between pediatric chronic pain with negative psychological characteristics such as depression and anxiety, little is known about the relationship of pediatric chronic pain with positive psychological characteristics such as emotional intelligence and self-esteem. The present study investigated three main questions: 

First, we assessed the prevalence of chronic pain among adolescents in Shiraz, Iran. We also compared emotional intelligence, self-esteem and parenting style between adolescents with chronic pain and healthy adolescents. Finally, the predicting role of emotional intelligence, self-esteem and parenting style in chronic pain was examined among adolescents.

## MATERIALS AND METHODS

This cross-sectional study, from January to June 2018, was conducted on adolescents (12-19 years old) in Shiraz, Iran. Assuming that at least 27% of adolescents in Shiraz suffer from chronic pain, ^17^ considering power: α: 5%, p: 0.27, q: 0.73, e: 0.025, and using the Cochran formula, we determined about an 800-subject sample size for the study.

$n_{0}=\frac{Z^{2}\mathrm{pq}}{e^{2}}$

Where:

e is the desired level of precision (i.e. the margin of error),

p is the (estimated) proportion of the population which has the attribute in question,

q is 1 – p.

A multistage clustering sampling method was used to select a representative sample of adolescents in Shiraz. Adolescents with chronic pain were identified by affirmative answers to three screening questions: (a) “Are you currently troubled by pain or discomfort, either all the time or on and off?” (b) Have you had this pain or discomfort for more than 3 months?” (c) “Does it affect your life and activities?” Adolescents with “yes” answer identified the main pain concern (defined as the worst pain and reported as the main reason for seeking treatment). Adolescents were also asked if they had been diagnosed with any common causes of pain (e.g., rheumatoid arthritis, migraine headaches, injury, etc). The assessment tool contained a numerical rating scale ranging from 0 (no pain) to 10 (worst imaginable pain) to assess the average intensity of pain during the last 2 weeks. The adolescents also provided information on the frequency (answer categories: permanent, one or more attack per day, one or more attack per week, one or more attack per month) and history of their pain (the number of months since they experienced pain). 

These items were developed based on the International Classification of Diseases 11th Revision (ICD-11) criteria. ^2^ The evaluation of the qualitative face validity was based on the opinions of 15 experts in the fields of nursing, anestesiology and pain medicine, to find any vague or irrelevant questions, or any difficulty with understanding of the questions. 89% of the experts recognized the questions as understandable and clear. To determine the quantitative face validity of each item, the item impact score was calculated. The impact scores showed that all the questions had a score equal to or greater than 1.5. Thus, in terms of face validity all the items were approved. Content validity was also evaluated qualitatively and quantitatively. The content validity index (CVI) for the items was between 0.87 and 1 that was above the required level to be acceptable. The content validity ratio (CVR) was also higher than 0.82; hence, the content validity has been approved. To examine test-retest reliability, the first 80 respondents answered the same questions for a second time, two weeks later. All questions showed correlation coefficients ≥0.74. 

This study was carried out in the largest region of Shiraz, a city in southern Iran. We drew a cluster sample of 16 randomly selected schools. First, all primary and secondary schools in Shiraz were stratified into 4 strata according to the delivery zones. Four schools (two primary and two secondary schools) were randomly (using simple random sampling) selected from each stratum. Then, in each school, we listed all the students at each grade. After that, 8-9 students were randomly (using systematic random sampling) selected at each grade. The inclusion criteria were: age 12-19 years old; student at one of the primary or secondary schools in Shiraz; capable of self-completing the questionnaires, and the ability to comprehend Persian language. Adolescents were excluded if they reported a history of chronic physical diseases not related to pain (e.g. asthma) or mental chronic disorders. 

800 adolescents and their parents were invited to participate in the study. The adolescents received a letter with information about the study, self-report instruments and consent forms for them and their parents to sign. The adolescents’ instruments and forms were completed in the classroom during school hours. The researcher was present and assisted the adolescents, if necessary. The parents’ questionnaire regarding parenting style and consent form were filled in by the most knowledgeable parent at home and returned to the teachers/ schools during 2 days. 

The study was approved by the Ethics Committee of the Shiraz University of Medical Sciences (IR.SUMS.REC.1396.S795). All participants were informed about the research project, the confidentiality of any disclosed information, and optional withdrawal from the study. Written informed consent form was signed by all the adolescents and parents who willingly participated in this study. The parents’ questionnaires were coded using numbers identical to those used to code the questionnaires of the adolescents. The data were collected anonymously without name lists. We attempted to increase the participation of schools, students and their parents by explaining the aims of the study. 

In addition to the demographic assessment (age, gender and birth order of adolescents, parents’ job and educational level), the other pertinent variables were assessed. Emotional Intelligence was assessed by the Trait Emotional Intelligence Questionnaire–Adolescent Short Form (TEIQue-ASF). ^18^ This questionnaire is one of the most common instruments for assessing emotional intelligence of adolescents. It contains 30 items. The items are responded on a 7-point Likert scale (1=completely disagree, 7=completely agree). The TEIQue-ASF score can range from 30 to 210. Higher scores indicate more emotional intelligence. ^18^ In terms of the psychometrics properties of the TEIQue-ASF, the internal consistency reliability of the scale on a sample of pupils was 0.84. ^19^ In a principal components factor analysis, all the items were loaded significantly on four factors and explained 69% of the variance in the measure. ^19^ In a study by Bayani, ^20^ the construct validity of the Persian version of the TEIQue-ASF was assessed by correlating its total score with the Oxford Happiness Inventory (r=0.59) and the School Burnout Inventory (r=-0.34) in a sample of students. The Persian version of the scale demonstrated good internal consistency (Cronbach’s alpha=0.78) ^20^ ; therefore, it has an optimum reliability and validity in Iranian population. 

Self-esteem was assessed by the Rosenberg self-esteem scale (RSES). ^21^ This scale consists of 10 items answered on a four-point scale ranging from 1 (strongly agree) to 4 (strongly disagree). The sum score for self-esteem varies from 10-40. Higher scores indicate more self-esteem. ^21^ The alpha reliability coefficient for the total scale was 0.93. Factor analysis revealed one factor, accounting for 44.8% of variance in test scores. The convergent validity of RSES was confirmed by strong positive correlations between RSES and other scales that were intended to measure global self-esteem such as Coopersmith Self-Esteem Inventory (r=0.68). ^22^ In a study by Rajabi and Bohlol, the construct validity of the Persian version of the Rosenberg self-esteem scale was assessed by correlating its total score with the Death Obsession Scale (r=-0.34) in a sample of students. ^23^ The Persian version of scale demonstrated good internal consistency (Cronbach’s alpha=0.84). ^23^

Baumrind parenting style questionnaire was used to assess the parenting style. ^13^ This questionnaire has 30 items. 10 out of 30 items are related to permissive style; 10 items are related to authoritarian style and 10 to authoritative style. All items are answered on a 5-point Likert scale and three separate scores were achieved by summing the scores of the questions of each style. ^13^ In terms of the psychometrics properties of the questionnaire, the following Cronbach coefficient alpha values were obtained for each of the styles: 0.75-0.87 for permissive style, 0.85-0.87 for authoritarian style, and 0.82-0.85 for authoritative style. The construct validity of the questionnaire was confirmed by significant correlations between Social Desirability Scale score with permissive style (r=0.23), authoritarian style (r=0.14), and authoritative style (r=0.10). ^24^ Farahini et al. showed the reliability of the Persian version of this questionnaire. Reliability coefficients of permissive style, authoritarian style and authoritative style were (Cronbach’s alpha=76%), (Cronbach’s alpha=72%), and (Cronbach’s alpha=74%), respectively. ^25^ In a principal components factor analysis, all items were loaded significantly on three factors and explained 30.47% of the variance in the measure. ^26^

Data were analyzed using SPSS software Version 22. In the beginning, compliance test for normal distribution with Kolmogorov–Smirnov test was applied. Levene’s test was used to examine the heterogeneity of the variances. Chi-square and Mann-Whitney tests were used to compare the differences in demographic and psychofamilial variables between adolescents with chronic pain and healthy ones. The method of binary logistic regression (method: Enter) assessed the association of every potential predictor (independent variable) with chronic pain in adolescents after controlling for demographic variables, as some studies have indicated these factors are correlated with emotional intelligence and self-esteem in adolescents. ^16 , 27 , 28^ Independent variables included emotional intelligence, self-esteem, authoritarian parenting style, authoritative parenting style, and permissive parenting style. The significance level was set at P<0.05.

## RESULTS

Of the 800 invited adolescents, 61 (7.62%) did not return the self-report instruments or consent forms, leaving 739 adolescents, with an overall response rate of 92.37%. Five adolescents had to be excluded from the study because they experienced a chronic disease not related to pain (such as asthma). Overall, 734 adolescents remained in the study. 

**Figure 1:**
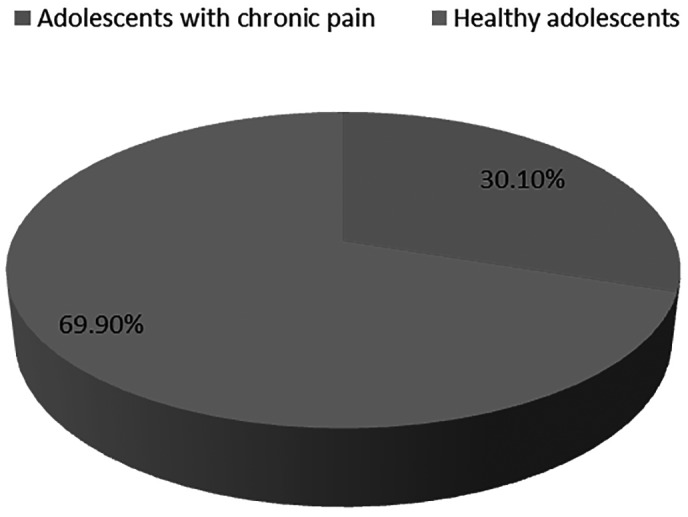
The frequency of chronic pain in adolescents.

The mean±SD of age of the adolescents was 14.95±1.36 years and the majority of them were girls 459 (62.5%). There were 221(30.1%) adolescents who met the ICD-11 criteria of chronic pain (Figure 1). The mean pain intensity over the two weeks was 3.2±0.61 on an 11-point numeric rating scale. They had a history of pain with a mean of 13±0.45 months; approximately one-third of adolescents with chronic pain had one or more pain attacks per month. Headache was the most frequently reported pain 74 (33.7%), representing approximately one-third of adolescents with chronic pain. Other commonly reported body locations included abdominal pain 48 (21.7%), spinal column pain 46 (20.6%), musculoskeletal pain 44 (20%), and other pain 9 (4%), respectively.

The results of chi-square analyses revealed that there were no statistical significant differences between adolescents with chronic pain and the healthy ones regarding the demographic variables, except for father’s job (P=0.004) (Table 1). 

**Table 1:** Comparison of demographic variables between the adolescents with chronic pain and healthy ones

Demographic variables		Adolescents with chronic pain (n=221) N (%)	Healthy adolescents (n=513) N (%)	X^2^	P value
Gender	Female	145 (65.61)	314 (61.21)	1.27	0.26
	Male	76 (34.39)	199 (38.79)		
Birth order	1	103 (46.60)	240 (46.78)	0.06	0.99
	2	65 (29.41)	143 (27.88)		
	3	31 (14.03)	80 (15.59)		
	4+	22 (9.96)	50 (9.75)		
Mother’s Education	High school or less	87 (39.37)	177 (34.50)	3.56	0.31
	Diploma	100 (45.25)	230 (44.83)		
	Bachelor	30 (13.58)	95 (18.52)		
	M.Sc./ Ph.D4 (1.80)	11 (2.15)			
Father’s Education	High school or less	82 (37.10)	164 (31.97)	3.91	0.27
	Diploma	87 (39.37)	190 (37.04)		
	Bachelor	42 (19.00)	123 (23.98)		
	M.Sc./ Ph.D10	(4.53)	36 (7.02)		
Mother’s Job	Worker/employer	32 (14.48)	71 (13.84)	0.81	0.66
	Retired	3 (1.36)	14 (2.73)		
	Unemployed	186 (84.16)	428 (83.43)		
Father’s Job	Worker/employer	185 (83.71)	418 (81.48)	11.17	0.004
	Retired	22 (9.95)	79 (15.40)		
	Unemployed	14 (6.34)	16 (3.12)		

Because Levene’s tests were significant and, therefore, equal variances were not assumed, Mann-Whitney tests were used to compare the differences in psychofamilial variables between the adolescents with chronic pain and healthy ones. Results of Mann-Whitney tests showed that emotional intelligence (P<0.001) and self- esteem (P<0.001) in healthy adolescents were significantly higher than those with chronic pain. As shown in Table 2, the score of authoritative parenting style of healthy adolescents was significantly higher than those with chronic pain (P=0.004). Also, the score of authoritarian parenting style was significantly higher in adolescents with chronic pain than healthy adolescents (P=0.006). There was no statistically significant difference between adolescents with chronic pain and healthy ones regarding the permissive parenting style (P=0.92) (Table 2). The current results clearly showed that there were significant differences regarding parenting style between the adolescents with chronic pain and healthy ones.

**Table 2:** Comparison of psychofamilial variables between the adolescents with chronic pain and healthy ones (n=734)

Variables		Adolescents with chronic pain (N=221) (Mean±SD)	Healthy adolescents (N=513) (Mean±SD)	U	P value
Emotional intelligence		132.13±28.68	147.21±24.59	38753	0.003
Self-esteem		29.86±6.25	32.48±5.04	43745	<0.001
Parenting style	Authoritative style	40.86±5.69	42.30±4.90	42124	0.001
	Authoritarian style	27.19±6.14	25.76±5.94	42405	0.004
	Permissive style	26.87±5.71	26.92±5.58	47877	0.92

### *The Predicting Role of Emotional Intelligence, Self-Esteem and Parenting Style in Chronic Pain among Adolescents*

Binary logistic regression revealed that emotional intelligence (P<0.001), self-esteem (P<0.001), authoritative parenting style (P=0.01) and authoritarian parenting style (P=0.04) were significant predictors of chronic pain in adolescents after controlling for demographic variables, but permissive parenting style (P=0.47) was not (Table 3).

**Table 3:** Logistic regression analysis: the association of emotional intelligence, self-esteem and parenting style with chronic pain (n=734)

Variables	Unadjusted			Adjusted^a^		
	OR^b^	95% CI^c^	P value*	OR^b^	95% CI^c^	P value*
Emotional intelligence	1.02	[1.01-1.02]	<0.001	1.02	[1.01-1.02]	<0.001
Self-esteem	1.08	[1.05-1.11]	<0.001	1.07	[1.04-1.10]	<0.001
Authoritative parenting style	1.05	[1.02-1.08]	<0.001	1.04	[1.01-1.08]	0.01
Authoritarian parenting style	0.96	[0.93-0.98]	0.005	0.97	[0.94-0.99]	0.04
Permissive parenting style	1.00	[0.97-1.03]	0.92	0.99	[0.96-1.02]	0.47

a adjusted for age, sex, birth order, mother’s education, father’s education, mother’s job, father’s job

b odds ratio (chronic pain/ healthy group)

c confidence interval

* Binary logistic regression, Reference group in regression analysis: healthy adolescents

## DISCUSSION

The present study showed that the emotional intelligence and self-esteem in healthy adolescents were significantly higher than those with chronic pain. The mean scores of authoritative and authoritarian parenting styles were significantly higher in healthy adolescents and those with chronic pain, respectively. Emotional intelligence, self-esteem, authoritative and authoritarian parenting styles were significant predictors of chronic pain in adolescents when controlling for demographic characteristics. 

Healthy adolescents showed higher levels of emotional intelligence compared to those with chronic pain. Emotional intelligence was also an important predictive factor of chronic pain in the present study. These findings are supported by other studies reporting that emotional intelligence is associated with physical health in adolescents. ^29 , 30^ According to literature, there are different ways in which emotional intelligence might influence pain in adolescents. For example, a review study concluded that higher levels of emotional intelligence may reduce pain vulnerability or pain experience through reducing high emotionality or negative mood, both known vulnerability factors for the development of chronic pain. ^29^ Some other studies have shown that adolescents with higher levels of emotional intelligence may better manage their stress. ^7 , 8^ It is well known that stress could negatively affect chronic pain in adolescents. ^9^ A recent study has also suggested that emotional intelligence contributes to the improvement of one’s health consciousness, and consequently participation in behaviors that predict long-term physical health. ^31^ In sum, although our findings do not shed light on causal relationships, the association between lower levels of emotional intelligence and chronic pain in adolescents is clearly shown. According to our results, emotional intelligence may be an important factor of the development or maintenance of chronic pain. It may emphasize the need for strategies to increase emotional intelligence in adolescents. Additional research is required to determine whether training in emotional intelligence could provide direct symptom relief or even potentially serve as a protective factor, reducing pain vulnerability in adolescents who may have other identified risk factors for the development of chronic pain.

The current results demonstrated a higher level of self-esteem in healthy adolescents than did adolescents with chronic pain. The results of logistic regression analyses showed that self-esteem was a significant predictor of chronic pain in adolescents. These findings are in the same line with those of some studies suggesting that children and adolescents with chronic illness have less self-esteem than their healthy peers. ^32 , 33^ The association between self-esteem and chronic pain may be explained by several mechanisms. For example, a lower level of self-esteem is shown to be associated with higher levels of anxiety and depression in adolescents, ^10^ both of which being associated with chronic pain. ^11^ Lower level of self-esteem is also associated with a lower level of resilience. ^34^ Resilience empowers adolescents to foster their skills and strengths to positively adapt and live successfully with their experiencing pain. ^34^ Adolescents with chronic physical illness who have higher self-esteem and high-quality friendships strive for positive and constructive life goals which consequently affect their well-being. ^32^ In contrast, self-esteem could be negatively affected by chronic illness, as chronic illness could affect the adolescents’ relationship to family and friends. ^32^ However, adolescents with chronic illness need to form close and reciprocal relationships with their family and friends to increase intimacy and emotional support, as well as confidence regarding managing their illness. ^32^ Although the direction of the relationship between chronic pain and self-esteem cannot be determined by this study, based on our results, there is a negative association between self-esteem and chronic pain in adolescents. These results could help health care providers such as pediatric nurses, psychiatric nurses or community health nurses to promote the holistic health of adolescents and prevent the pain from becoming a chronic problem in them. In this way, assessment and education of psychological skills should be included as routine core elements in the integrated care of adolescents by nurses.

The mean score of authoritative parenting style in healthy adolescents was higher than those with chronic pain. Moreover, the mean score of authoritarian parenting style in adolescents with chronic pain was higher than the healthy peers. In line with the results of t-tests, regression analyses also reflected that authoritative and authoritarian parenting styles significantly predicted chronic pain in adolescents. In accordance with our results, previous studies showed that there was a reverse relationship between parenting authoritative style and physical complaints, and a positive relationship between authoritarian parenting style and physical complaints. ^15^ Anno et al. revealed that high levels of overprotection were associated with an increased risk of chronic pain. ^35^ Also, it has been suggested that adverse experiences during childhood are possible risk factors for the development and persistence of chronic pain. ^35^ Some studies provided evidence that dysfunctional parenting styles were associated with a lower level of self-esteem, ^36^ more dysfunctional interpersonal relationships, ^15^ and poorer coping with stress, ^37^ all known as predictors of chronic pain. Interestingly, there was no statistically significant difference between adolescents with or without chronic pain regarding the permissive parenting style. Also, binary logistic regression analysis did not reveal a significant correlation between permissive parenting style and chronic pain. Consistently, one study showed that mental health problems in adolescents were associated more with authoritarian and less with permissive patenting style. ^15^ One other study demonstrated similar results about the association of parenting styles and depression among adolescents. ^38^ This finding raise the possibility that parenting styles characterized by low care and high overprotection make adolescents more susceptible to chronic pain than parenting styles characterized by low care and low overprotection. To sum up, our findings suggest that maladaptive parenting styles, especially those characterized by low care and high overprotection (authoritarian parenting style), could be related to the risk of development of chronic pain in adolescents. Therefore, education on parenting behaviors for optimal parenting style may be one of the most important initiatives for pediatric health care providers such as pediatric nurses, psychiatric nurses or community health nurses. However, further studies are required to identify the mechanisms whereby parenting styles affect the development of chronic pain in adolescents.

It is important to mention some of the limitations of our study. Chronic pain was assessed based on the self-report questionnaire and not on clinical examination. Compared to clinical assessment, the accuracy of self-report measures may be affected by response bias. ^39^ Further, given the cross-sectional design, our findings do not shed light on causal relationships. Prospective longitudinal studies are needed to clarify the contribution of psychological characteristics of adolescents and the parenting styles to the development of chronic pain. Moreover, undoubtedly there are factors other than those assessed in this study that also affect the chronic pain in adolescents. Therefore, studies assessing other psychofamilial variables not assessed in this study are recommended. 

## CONCLUSION

 Our findings highlight that emotional intelligence and self-esteem might reduce pain vulnerability in adolescents. Moreover, maladaptive parenting styles could be related to the risk of development of chronic pain in adolescents. Although correlational results do not shed light on causal relationships, our findings add some evidence to further support the influence of psycho-familial factors on chronic pain in adolescents. Therefore, the most effective preventive program for adolescents at risk of chronic pain requires a comprehensive biopsychosocial understanding of pediatric chronic pain. If these results are confirmed by interventional studies, interventions improving psychological and parental skills are important in terms of preventing development of chronic pain in adolescents. The current results could help clinicians, parents and teachers to determine which factors should be emphasized in order to prevent the development of chronic pain in adolescents.
